# Overcoming barriers to facilitate the regulation of multi-centre regenerative medicine clinical trials

**DOI:** 10.1186/s13287-018-1055-2

**Published:** 2018-11-08

**Authors:** Erika Kleiderman, Audrey Boily, Craig Hasilo, Bartha Maria Knoppers

**Affiliations:** 1 Centre of Genomics and Policy, Department of Human Genetics, McGill University, Montreal, QC, H3A 0G1 Canada; 2 CellCAN, Pavillon Rachel-Tourigny RT2101, Montreal, QC, H1T 2M4 Canada

**Keywords:** Ethics, Policy, Regulation, Regenerative medicine, Multi-centre clinical trial, Cell therapy

## Abstract

In the context of regenerative medicine and cellular therapies, the treatment under study often targets a less common disease or condition for which recruitment of a large number of research participants at any given site is challenging, if not impossible. One way to overcome this challenge is with a multi-centre clinical trial. This manuscript first aims to briefly outline the existing ethical, legal and social implications as well as the regulatory frameworks associated with multi-centre regenerative medicine clinical trials. Second, it considers the regulatory limitations and barriers surrounding the initiation of such trials in Canada, the USA and Europe. Third, it concludes with a set of recommendations for facilitating multi-centre clinical trials, at both national and international levels.

## Background

Regenerative medicine can be described as a “field of clinical research and application that seeks to use biological materials and mechanisms to repair, restore, maintain or improve the function of tissues and whole organs” (p. 2) [1]. Cell-based therapies in the clinical research pipeline pass through stringent safety testing including dosing trials for efficacy and eventually into larger cohorts of patients in clinical trials with the aim of becoming approved therapies. Once approved by a regulatory body for market authorization (e.g. Health Canada; US Food and Drug Administration (FDA); European Medicines Agency (EMA)), cell-based therapies can be legally offered. In short, this regulatory approval process takes time.

In recent years, there has been an influx of unapproved cell-based therapies making it to market in Canada and elsewhere via private clinics [2]. This is problematic, as unproven stem cell therapies are neither approved nor regulated, are offered directly to patients on a fee-for-service basis often costing thousands of dollars, and potentially endanger the “patients”. Furthermore, the marketing of these unproven therapies often preys on the hope for a cure or the alleviation of disease, despite the lack of evidence of safety and efficacy. Any cell-based therapy regulated as a drug (discussed below) cannot be provided outside of properly controlled clinical trials until the drug has obtained full market approval.

Since the early 2000s, the number of cell-based therapy clinical trials has steadily increased with a global revenue stream of more than $1 billion USD [3, 4]. Our understanding of cellular therapies and regenerative medicine has progressed over the past decade with improvements in safety, efficacy and delivery of technologies for both research and therapeutic purposes. Their clinical potential for a range of diseases is promising, albeit, with ongoing limitations as to their successful clinical translation and uptake [5]. Clinical activity appears to be “clearly converging upon a critical mass, with over 300,000 patients treated with regulatory-approved products since 1997” (p. 49) [4], and almost 977 regenerative medicine clinical trials (RMCT) currently underway around the world [6]. This highlights the need to manage increasing expectations to both avoid hype and, ultimately, disappointment in the field [4].

There is no doubt that traditional regulatory frameworks require adaptation or may no longer be suitable to align with and address the unique safety, efficacy and quality issues as regenerative medicine moves towards clinical trials [7, 8]. Better cross-jurisdictional infrastructure for clinical trials would accelerate the development and clinical translation of efficient cell and gene therapies [9]. Indeed, as suggested by the 2018 OECD report entitled *Gene editing for advanced therapies: Governance, policy and society*, the “creation of a permanent, but flexible, infrastructure that work[s] to speed up the trials process, while not sacrificing safety and efficacy data collection required by regulators, could provide meaningful benefits—including critical mass—to the innovation process” (p. 20) [9].

The traditional phased model approach to clinical trials is complex and cumbersome especially during phase III efforts that aim to compare and evaluate the effectiveness of new treatments with those currently available [10, 11]. A substantial number of research participants is required. Yet, in the context of regenerative medicine and cellular therapies, the treatment under study often targets a less common disease or condition for which recruitment of a large number of research participants at any given site is challenging, if not impossible [11, 12]. One way to overcome this challenge is with a multi-centre clinical trial, which entails “a controlled study executed by several cooperating institutions” (p. 21) [13]. For example, the Mesenchymal Stem cell therapy for Canadian Multiple Sclerosis patients (MESCAMS) study, a multi-centre clinical trial, is the first in Canada to evaluate the safety and efficacy of mesenchymal stem cells to treat multiple sclerosis. Part of a larger, international research undertaking entitled Mesenchymal Stem Cells for Multiple Sclerosis (MESEMS), MESCAMS helps to facilitate the pooling of resources and access to expertise from nine countries all undertaking similar research, which in turn accelerates discovery and the generation of conclusions [14]. Another Canadian example of an active multi-centre clinical trial is the Canadian National Transplant Research Program’s CARE Trial, which assesses the clinical efficacy of continuous alloreactive T cell depletion and regulatory T cell expansion in steroid-refractory or dependent chronic graft versus host disease [15]. It includes sites in four provinces (British Columbia, Manitoba, Ontario and Quebec).

Although more costly and time-consuming to set up than single-centre clinical trials, a multi-centred approach provides many advantages. These include a larger and more heterogeneous sample of participants for more robust statistical analyses, a broader basis for generalization and the pooling of protocols, costs and personnel [16]. Before undertaking a multi-centre clinical trial, however, the following must be considered: proof that multiple centres are needed to meet the requirements for sample size outlined by the study, an identifiable group of clinician-researchers who agree to collaborate and abide by a common research protocol and an identifiable group of clinics with the necessary resources to conduct the trial (i.e. resources, personnel, facilities) [10, 16].

In sum, gaps exist in the advancement of multi-centre RMCTs. There is a need and an interest in facilitating collaborations so as to bridge these gaps and collectively learn from the experiences of others [9]. This manuscript first aims to briefly outline existing ethical, legal and social issues (ELSI) as well as the regulatory frameworks associated with multi-centre RMCTs. Second, it considers the regulatory limitations and barriers surrounding the initiation of RMCTs in Canada, the USA and Europe. Third, in conclusion, it proposes a set of recommendations for facilitating multi-centre RMCTs, at both national and international levels.

## Ethical, legal and social issues

Many of the ELSI span the entire spectrum of the translation process from basic stem cell research and clinical trials to access to attendant medical and therapeutic technologies (Table 1). In the context of basic research, one of the primary concerns deals with the quality and integrity of the research being conducted along with the transparency/publication of research results that impact public trust [1]. Moreover, an inherent tension arises between the pace of clinical progress, scientific caution and compliance with regulatory requirements [17].

**Table 1:** Key ELSI

Basic research	Clinical research	Clinical context
• Quality and integrity of research • Transparency and publication of results • Inherent tension between clinical progress, scientific caution and compliance with regulatory requirements	• Safety and efficacy of intervention (evidence-based) • Informed consent • Prohibitively high development costs • Time- and labour-intensive • Justice and affordability • Pressure to commercialize	• Unproven therapies • Right to try • Accuracy in advertising • Legitimacy of the informed consent obtained • Vulnerable population exposed to unjustifiable risks

Irrespective, within the context of clinical research, the safety and efficacy of the intervention under study must be evidence-based [18]. The ability to provide information and obtain proper informed consent from the patients who are to be part of the trial [19], as well as the primacy of their welfare [20], are crucial elements to consider. Past scandals and patient deaths [21] during the course of clinical trials have led decision-makers to readjust legislation and regulation governing regenerative medicine [22]. Safety issues may also impact public perceptions about the research enterprise and sway a participant’s willingness to participate in a given clinical trial [23].

A major objective of the clinical research is to develop a therapy that will receive market authorization and be offered to patients and future generations as a preventive or treatment option for years to come. As stem cell research is primarily conducted by academic researchers, issues related to its clinical translation may arise, as previously mentioned [24]. For example, a clinical research conundrum exists within Canada, as clinical trials and therapeutic development currently proceed under “clinical research”, even though they are used to generate safety data (i.e. early phase or phase I/II) prior to generating efficacy data (phase II/III). Moreover, the costs associated with the development of a regenerative medicine intervention can be prohibitively high as well as time- and labour-intensive [25]. This raises considerations of justice and affordability of the treatment, as well as priority setting and the possibility for government funding, if at all [22, 25]. Furthermore, there is the pressure placed on clinician-researchers for rapid clinical translation and continuing commercialization of the stem cell therapies under development [25, 26], which may in turn impact integrity [27]. Indeed, the general issues faced in all clinical research such as the return of results, informed consent, and privacy are part of RMCTs as well [28, 29]. Patients themselves are challenging these multiple requirements and ensuing delays.

Many patients facing life-threatening illness claim that there should be no barriers to access unproven therapies and advocate for the “right to try” [19]. This approach is underscored by the President of the USA signing into law the “right to try” bill on May 30, 2018. The law allows terminally ill patients to gain access to experimental treatments not yet approved by the US FDA [30]. Yet, debate continues as to the actual benefits of such a law [31]. In contrast, the Australian regulatory authority, the Therapeutic Goods Administration (TGA), has recently taken a stance against the current marketing and advertising of autologous human cell and tissue therapies. As of July 1, 2018, the TGA has banned advertisements of unproven therapies to consumers and requires the reporting of all adverse events [32, 33].

## Regulatory frameworks

Beyond these ELSI, the regulatory processes for the approval of RMCTs in Canada, the USA, and Europe vary. Each jurisdiction has its own regulatory agency involved in overseeing and managing clinical trial applications (CTA), respectively Health Canada, the US FDA, and the EMA. There are additional provincial or state requirements in Canada and the USA as well as additional national requirements in countries of the European Union. Both Health Canada and the US FDA “stratify somatic cell products based on whether they are [allogeneic], minimally manipulated, used in a homologous fashion, combined with other drugs and/or devices, and have systemic/metabolic effects” (p. 609) [34]. Below, we address cell and tissue products (CTPs) which are more than minimally manipulated and intended for non-homologous purposes. This type of CTP was selected because “most cell therapy products are likely to have some systemic and/or metabolic effect” (p. 650) [35]. Even though autologous stem cell products are not the subject matter of this paper, the findings from a recent Canadian workshop on the gaps in the regulation of minimally manipulated autologous stem cell therapies might be particularly instructive [36].

### Canada

In Canada, cell therapy products that are more than minimally manipulated and that neither meet the criteria outlined in the *Safety of Human Cells, Tissues and Organs (CTO) for Transplantation Regulations* (i.e. section 3(1)—that is they are allogeneic, minimally manipulated and used for homologous purposes) [37]—nor the criteria of the *Medical Devices Regulations (MDR)*, are regulated under the *Food and Drugs Regulations* (F&DR) as drugs (Table 2). Under section 3(1)d) of the *CTO Regulations*, tissue and cells “that have a systemic effect and depend on their metabolic activity for their primary function” are also typically excluded from the *Regulations*. Yet, islet cells and lymphohematopoietic cells derived from bone marrow, peripheral blood or cord blood have been exempted from this exclusion as evidence of their safety and efficacy has already been established through clinical trials and practice [37]. Divisions of the F&DR that find application are divisions 1 (General), 1A (Establishment Licenses), 2 (Good Manufacturing Practices), 4 (Schedule D Drugs (Biologics)), 5 (Drugs for Clinical Trials Involving Human Subjects) and 8 (New Drugs) of part C (Drugs). However, it is mostly division 5 that finds direct application for clinical trials in humans [35]. It stipulates what criteria must be met in Canada to sell or import a drug for the purpose of a clinical trial. As such, there is not only a requirement for an application for clinical trial authorization or amendment, but also the sponsor’s obligation to Good Clinical Practices, to label, record and report adverse drug reactions.

**Table 2:** Key definitions (Canada)

Safety of Human Cells, Tissues and Organs (CTO) for Transplantation Regulations, Article 1	
Homologous in respect of a cell, tissue or organ, means that the cell, tissue or organ performs the same basic function after transplantation.	
Minimally manipulated means	
(a) In respect of a structural tissue, that the processing does not alter the original characteristics that are relevant to its claimed utility for reconstruction, repair or replacement; and (b) In respect of cells and non-structural tissue, that the processing does not alter the biological characteristics that are relevant to their claimed utility.	
Food and Drugs Act, Article 2	
Drug includes any substance or mixture of substances manufactured, sold or represented for use in	
(a) The diagnosis, treatment, mitigation or prevention of a disease, disorder or abnormal physical state, or its symptoms in human beings or animals, (b) Restoring, correcting or modifying organic functions in human beings or animals, or (c) Disinfection in premises in which food is manufactured, prepared or kept.	
[emphasis added by the authors]	

Cell therapy products that are subject to the F&DR must seek specific Health Canada authorization to proceed with a clinical trial [35]. In order to assist clinician-researchers and industry with the interpretation of part C, division 5 of the F&DR, the Government of Canada has developed a Guidance Document to clarify the authorization process [38]. Generally, to acquire an authorization, a clinician-researcher or sponsor must submit a CTA for each phase of the clinical trial process. The CTA needs to provide detailed information about the clinical trial protocol, manufacturing/clinical information about all unapproved components and components not approved for that use, Research Ethics Board (REB) approvals and Good Laboratory/Clinical Manufacturing Practices compliance. Authorization requires evidence that the clinical trial does not endanger the interests of the participants. As the clinical trial status progresses from early to late phase (phase I/II versus III/IV), additional details are required for batch record keeping documenting the manufacturing process. The CTA is examined within 30 days of receipt by Health Canada, following which one of three responses is provided: (1) “no-objection” letter (i.e. approval), (2) further clarifications are required prior to approval (i.e. conditional approval) or (3) non-satisfactory notice (i.e. rejected). The clinician-researcher or sponsor then has 30 days to resubmit [35]. For market approval and authorization of a drug, clinician-researchers or sponsors can submit new drug submissions to Health Canada, according to division 8, part C of the F&DR [35]. That being said, prospective clinician-researchers and sponsors should nevertheless remain mindful of the environmental impact of substances used in clinical trials. These may, under certain conditions, trigger the New Substance Notification Regulations under the *Canadian Environmental Protection Act*.

### USA

In the USA, human cell and tissue-based products (HCT/Ps) that do not meet all of the criteria outlined in the 21 *Code of Federal Regulations* 1271.10(a) (i.e. more than minimally manipulated and/or intended for non-homologous use) are typically regulated under both sections 351 and 361 of the *Public Health Services Act* (PHSA) [39]. The FDA has jurisdiction over HCT/Ps, which are considered to be drugs and/or biological products [39]. Section 351 PHSA authorizes the FDA to attribute licenses for drugs and/or biological products, while Section 361 PHSA mandates the FDA “to issue and enforce regulations necessary to prevent the introduction, transmission, or spread of infectious disease” (p. 955) [40]. Regulation of HCT/Ps is carried out by the Center for Biologics Evaluation and Research’s (CBER) Office of Cellular, Tissue, and Gene Therapies (OCTGT) within the FDA [39]. Clinician-researchers or sponsors must submit an application to obtain an Investigational New Drug (IND) designation in order to conduct clinical trials [39]. Once clinical trials are complete, the FDA controls market entry with specific requirements for pre-market testing of safety and efficacy [39]. As such, the selected HCT/Ps will require pre-market authorization from CBER and must comply with Good Manufacturing Practices (GMPs) [39]. The FDA’s mandate has been expanded to include facilitating innovation and expediting the development of new therapies, notably in the context of serious or life-threatening diseases [40]. This was clearly articulated in the *Twenty-first Century Cures Act* (2016) [41], which introduced a new accelerated pathways program to expedite the development and review of regenerative medicine advanced therapies (RMAT). RMAT designated HCT/Ps and cell therapies must demonstrate preliminary evidence of their potential to address unmet medical needs related to the serious or life-threatening disease, and once approved by the FDA, such designated products may be eligible for priority review and accelerated approval [40]. Furthermore, the FDA has released a comprehensive oversight framework laid out in four guidance documents that clarify the criteria, definitions and interpretations for the advancement of regenerative medicine [40, 42, 43].

### Europe

In Europe, advanced therapy medicinal products (ATMPs) are either gene therapy medicinal products, somatic cell therapy medicinal products or tissue-engineered products [44]. Such products are typically subject to *Regulation (EC) 1394/2007 on Advanced Therapy Medicinal Products* (adopted in 2007) [45, 46], which was supported by *Clinical Trials Directive 2001/83/EC* (repealed) and *Regulation (EC) No 726/2004* [47]. Furthermore, clinician-researchers and sponsors must also conform to the general framework set out in the new *Clinical Trials Regulation 536/2014*, which puts forward a single CTA approach, for all Member States involved in a given proposal, via a single online EU portal and the possibility to provide greater transparency in the reporting of data post clinical trial [47, 48]. *Regulation (EC) 1394/2007* also sets out additional criteria for ATMPs and creates a centralized authorization process for those marketed within the European Union (EU) [39]. The additional criteria lead to a more “rigorous” evaluation of ATMPs than was the case under previous medicinal product legislation [49]. As a result, this centralized process may result in longer delays in authorization [22].

In the case of doubt regarding the classification of an ATMP, the EMA has established an optional procedure through which a clinician-researcher or sponsor can submit an application for a scientific recommendation on the classification of ATMPs [49, 50]. To qualify as an ATMP, the cells or the tissues must undergo a process that “involves substantial manipulation of the starting materials” (p. 426) [49]. The Committee for the Advanced Therapies (CAT) is responsible for all regulatory procedures concerning ATMP in the EU (p. 410) [51], notably “the adoption of scientific recommendations on ATMP classification taking in to account the legal provisions in force, the scientific state of the art and the input from the European Commission” (p. 4) [50]. If a product is qualified as an ATMP, the clinician-researcher or sponsor will need to obtain marketing authorisation (MA) by submitting a file containing all the data collected during the product development phase that demonstrates “the quality, safety and efficacy of ATMPs” (p. 427) for market approval [49]. Guidance documents are available to further describe the specifics for safety, quality and efficacy aspects [49].

## Limitations and barriers to initiating multi-centre regenerative medicine clinical trials

The ELSI highlighted above, along with the regulatory frameworks themselves, raise barriers and limitations for the initiation of multi-centre RMCTs (Table 3). Authors have also noted a lack of international coordination in the harmonization of regulatory requirements for RMCT [52]. This makes the complex and diversified regulatory landscape difficult, to say nothing of creating variation and incompatibility between collaboratory centres from different jurisdictions. Regulatory approval processes are difficult to navigate, notably in the context of multi-centre clinical trials.

**Table 3:** Key limitations and barriers

Lack of international coordination	• Complex and diversified regulatory landscape • Variations and incompatibilities
Uncertainty and lack of clarity in regulations	• Difficult to read and understand • Lack specificity and clarity
Organizational complexity from interactions with medical authorities and regulatory entities	• Information available online not always current or user-friendly • Requirements vary by country and regulatory authority
Costs and infrastructure requirements	• Lack compatible infrastructure across clinical trial sites • Funding and disproportionate financial burden (academia)
Cultural and logistical disparities	• Variations in ethical (e.g. REB approval), confidentiality and privacy requirements from country to country
Unproven therapies	• Small number of approved cellular therapies on the market • High price tag • Gaps in oversight and enforcement of such mechanisms

Notwithstanding, there are a number of international harmonization initiatives such as the International Council on Harmonization (ICH), International Pharmaceutical Regulator’s Forum Cell Therapy Group, International Pharmaceutical Regulator’s Forum Gene Therapy Group, Pharmaceutical Inspection Cooperation Scheme (PIC/s) and Regional Cooperation Council (RCC), as well as an increase in collaborative efforts to address issues between Canada, the USA and the EU [53]. However, these initiatives may overlap and create a lack of coordination at another level, resulting in the ideal of a harmonized system being a long way off. Indeed, there appears to be a global shift towards regulatory diversification rather than harmonization [54]. According to the 2018 OECD report on advanced therapies, a decrease in the number of ongoing initiatives would minimize the duplication of efforts while better coordination could facilitate harmonization [9].

Uncertainty also exists regarding the regulations that are actually in place since accompanying documentation lacks clarity (e.g. guidelines, templates and approval processes) [55]. Yet, a counterargument to this concern would be that regulatory policy, by its nature, is “performance-based” and intentionally lacks specificity. Specific guidance is difficult to write and maybe restrictive in its application once created (i.e. more efficient to refer to general guidance). Thus, uncertainty in regulations may in fact allow for greater flexibility throughout the approval process (from clinical trials to post-market).

Another barrier is the organizational complexity resulting from interactions with medical authorities and regulatory entities in various countries [56]. The information that is available online is often not very user-friendly, time-consuming to navigate, and the websites are not current regarding regulatory requirements [55]. Revisions take significant time and resources to carry out and, however, are sporadic. Furthermore, there is a strong push for national databases containing the clinical data that is used to support the approval of drug and medical device applications. In Canada, for example, Health Canada is considering policy changes to begin making this information readily available to the public [57, 58]. Easier access to complete and up-to-date information is needed.

Furthermore, multi-centre RMCTs are costly and resource-intensive due to the required equipment and highly qualified personnel operating within the cell and gene therapy GMP facilities [12, 59]. The authors have noted a lack of compatible infrastructure across clinical trial sites (e.g. hospitals or institutions) [60] and the challenges of disseminating knowledge about the process for submitting CTAs [61]. Funding is always an important consideration as well, when a research project scales up to a multi-centre trial. Although the field has been driven by academic institutions and small- to medium-sized organizations, there is a disproportionate financial burden placed on these entities, as they typically lack the resources to deal with regulatory uncertainty and complex approval pathways (e.g. hire regulatory experts to guide them through the process), that big pharmaceutical companies possess [23]. One of the primary criticisms surrounding the current European *Clinical Trials – Directive 2001/20/EC* is its shortcomings in addressing the needs of academia versus industry (i.e. the increased costs and the regulatory complexity of procedures for academics) [62]. A counterargument to the disproportionate financial burden for academia would be that in Canada, federally funded Networks of Centres of Excellence (e.g. CellCAN, CCRM, C3i, BioCanRx) provide support and regulatory advice as a component of existing agreements with groups like Medicine by Design, OIRM, BC RegMed and ThéCell (i.e. free to researchers), while industry must maintain infrastructure and regulatory groups (i.e. out-of-pocket expenses). In this role, groups like CellCAN act as conduits between the Cells, Tissue and Viral Vector Manufacturing Facilities (CMF) stakeholders and regulators while providing an outlet for Health Canada to address regulatory concerns and clarifications around the expectations for CTAs. Perhaps then, a way to alleviate financial burdens would be to encourage academia to better prepare and organize so as to increase awareness of such services and ensure that research groups get the support they need. In the same vein, the substantial regulatory requirements surrounding clinical translation that burden academic institutions require careful advanced planning and proper attribution of resources to increase overall efficiencies in progressing through the clinical trials pipeline and meet the safety and efficacy criteria prior to approval and marketing [63].

Other factors intrinsically linked to international multi-centre trials in the context of regenerative medicine are cultural and logistical disparities between the different institutions involved. These issues must be considered when planning clinical trials and developing uniform procedures [22]. For example, the information to be transmitted to participants, as well as the quality and availability of infrastructures, vary from country to country [60]. Clinician-researchers should also be aware of different health insurance requirements and availability in different countries that may affect the financial sustainability of the research project [22]. Another challenge stems from obtaining REB approval and subsequently coordinating various agreements. The REB approval process, requirements and privacy issues are unique to each country and may vary by institution, which hinders multi-centre clinical trials if all sites are not able to obtain approvals in a timely manner. As well, site-specific issues may arise, for example, executing quality agreements between manufacturing sites.

Finally, as mentioned above, unproven therapies are on the rise [64]. This increase is reportedly the result of onerous regulations that have led to only a small number of approved stem cell therapies making it to the US market [29], with prohibitively expensive price tags [21]. Conversely, one may also argue that the regulations are not truly onerous but rather appropriately balance the potential risks of the therapy through an independent third party (i.e. drugs/products that do not meet regulatory standards should not be offered to the public). They act as a barometer to determine if a given drug/product has demonstrated enough evidence of safety to move towards the market; therefore, the critique should be on the difficulty in establishing this evidence that holds upmarket approvals and not on onerous regulations. Moreover, there is a gap in oversight and the enforcement of such mechanisms that often leads to exploitation [65]. Thus, efforts are being made to strengthen regulatory oversight and discourage clinics from marketing therapies that lack an evidentiary basis [66]. As outlined in the International Society for Stem Cell Research’s (ISSCR) 2016 *Guidelines for stem cell research and clinical translation*, “all research involving clinical applications of stem cell-based interventions must be subject to prospective review, approval and ongoing monitoring by independent human subjects review committees” (p. 19) [67]. This recognizes the value and importance of independent research oversight in ensuring the ethical conduct of research, the protection of human participants and the generation of credible data.

## Conclusions

We propose the following initiatives as a way of addressing and perhaps overcoming the limitations and barriers to initiating multi-centre RMCTs, from both national and international perspectives:

1. *International coordination* in the harmonization of existing regulatory requirements and pathways. A first step forward would be to focus on the clarification and standardization of the terminology currently used and outlined in the regulatory process, as there exist variations in the critical definitions used in cellular therapies (e.g. minimal manipulation, homologous versus non-homologous use) [24]. From there, a consensus strategy could be built to enable the harmonization and prioritization of tasks along with a push for global standards. Such a process will be time-consuming, as there is a need to adjust to varying criteria, which “necessitates far-reaching forms of scientific self-governance, training and procedural adjustments in participating clinical trial sites” (p. 305) [56]. An approach that consolidates or decreases the number of initiatives would enable the focus to be on improved and streamlined coordination.
2. *Oversight* could be provided by international authorities (e.g. ISSCR and the International Society for Cellular Therapy) so as to counteract the diversification of regulation, despite their non-binding nature.
3. Development of an *international approach for ethics approvals* of multi-centre clinical trials that build on the principle of mutual recognition [68, 69]. Such an approach could take the form of a submission template containing all required information common to each institution involved while allowing specific additions to be made by way of appendices. This would maximize transparency and lead to a more simplified and streamlined approach to REB approvals while reducing the duplication of efforts.
4. Creation of an interactive *online educational platform* for knowledge mobilization containing information on the regulatory requirements and procedures for CTAs on a large number of countries, so as to centralize and facilitate access to such information by researchers. Researchers from different countries looking to conduct multi-centre clinical trials could communicate and discuss the feasibility of proposed trials. For example, under the new *EU Clinical Trials Regulation* (*Regulation (EU) No 536/2014*), a publicly accessible EU database and portal will be created to act as a one-stop-shop for all CTAs [70]. This will be set up and managed by the EMA, European Commission and the Member States, thus addressing the diversity of infrastructure requirements and systems in place in different countries. Of note, however, is that the European system may be difficult to translate into either the Canadian or US contexts due to the lack of an equivalent authority.
5. *Initiate dialogue with regulatory entities* early in the process in order to collect all necessary information regarding criteria, safety and efficacy standards, and assure timely turnover of CTAs. This can be spearheaded by national regulatory authorities as they ensure that the scientific expertise and staffing needed to assure timely assessment of CTAs is in place. Organizations such as CellCAN and the ISCT North America Legal and Regulatory Affairs Committee have created meetings for open dialogue with regulators from each jurisdiction (Health Canada and the US FDA, respectively) that allow cell therapy stakeholders to concert efforts and address other pressing issues facing the RMCT sector with a united front. Furthermore, by establishing an advanced dialogue with regulatory agencies, basic researchers and clinician-researchers will be able to receive support, information and guidance early on.
6. *Clarification* and *re-examination* of the boundaries between research and innovation are necessary. Research comprises the necessary building blocks and foundational work for scientific breakthroughs. Yet, the path from research to innovation is evolving. This research branding of the clinical trials pipeline is not a new paradigm. The same held true for previous generations of ground-breaking cellular therapies such as bone marrow transplantation when it was first used in the clinic in the 1970s and now considered a routine medical procedure [71]. Although the traditional clinical trials model is still the most used approach, there are ways of making innovative therapies available (e.g. hospital exemption or compassionate treatment) [59]. As such, traditional boundaries must also be revisited in light of this evolution.

These initiatives are important because cell-based therapies hold promising clinical applications for the treatment and prevention of a wide range of diseases and conditions. Nevertheless, there remain important barriers and ELSI issues associated with initiating such trials that need to be addressed. As such, there is a need to propose a way to bridge the regulatory gaps so as to facilitate multi-centre RMCTs. This will enable collaborative efforts to collect the necessary safety and efficacy evidence by providing a larger sample of participants, while pooling resources (i.e. protocols, personnel and costs). Ultimately, this will favour the acceleration of cell-based therapies into the clinic.

## Abbreviations

ATMP: Advanced therapy medicinal products
CBER: Center for Biologics Evaluation and Research
CTA: Clinical trial application
CTO: Cells, tissues and organs
CTP: Cell and tissue product
ELSI: Ethical, legal and social issues
EMA: European Medicines Agency
F&DR: Food and Drugs Regulations
FDA: Food and Drug Administration
HCT/P: Human cell and tissue-based product
ISSCR: International Society for Stem Cell Research
MESCAMS: Mesenchymal Stem cell therapy for Canadian Multiple Sclerosis patients
PHSA: Public Health Services Act
REB: Research ethics board
RMAT: Regenerative medicine advanced therapies
RMCT: Regenerative medicine clinical trial
TGA: Therapeutic Good Administration
